# Multistable Protocells Can Aid the Evolution of Prebiotic Autocatalytic Sets

**DOI:** 10.3390/life13122327

**Published:** 2023-12-12

**Authors:** Angad Yuvraj Singh, Sanjay Jain

**Affiliations:** 1 Department of Physics and Astrophysics, University of Delhi, Delhi 110007, India; aysingh@physics.du.ac.in; 2 Santa Fe Institute, 1399 Hyde Park Road, Santa Fe, NM 87501, USA

**Keywords:** origin of life, protocells, autocatalytic sets, prebiotic evolution

## Abstract

We present a simple mathematical model that captures the evolutionary capabilities of a prebiotic compartment or protocell. In the model, the protocell contains an autocatalytic set whose chemical dynamics is coupled to the growth–division dynamics of the compartment. Bistability in the dynamics of the autocatalytic set results in a protocell that can exist with two distinct growth rates. Stochasticity in chemical reactions plays the role of mutations and causes transitions from one growth regime to another. We show that the system exhibits ‘natural selection’, where a ‘mutant’ protocell in which the autocatalytic set is active arises by chance in a population of inactive protocells, and then takes over the population because of its higher growth rate or ‘fitness’. The work integrates three levels of dynamics: intracellular chemical, single protocell, and population (or ecosystem) of protocells.

## 1. Introduction

The simplest life forms existing today and plausibly existing at the origin of life are such complex chemical organizations, involving small and large molecules, that it is virtually impossible to imagine their origin except through some process of chemical evolution [1,2]. Imagining plausible steps in chemical evolution that resulted in the increase in complexity of prebiotic chemical organization is, therefore, an important task.

One significant set of prebiotic scenarios is based on the idea of an autocatalytic set (ACS) of chemical reactions [3,4,5]; reviewed in [6,7]. Here, we are concerned about the evolution of ACSs. This has been investigated [8,9,10,11] (for reviews, see [12,13]) largely in the context of ACSs that reside in static well-stirred containers. It is recognized that at some stage autocatalytic networks must have evolved inside a spatial compartment or ‘protocell’ which propagated through growth and division. Consequently, different models of protocells containing ACSs have been proposed [14,15,16,17,18,19,20,21,22,23], where the compartments are modeled after micelles (autocatalytic aggregates of lipid catalysts), vesicles (lipid bilayers permeable only to food molecules enclosing an aqueous environment containing the ACS), or other structures.

These models have considered how the features of Darwinian evolution [24,25], namely, (i) heredity, (ii) heritable variation, and (iii) differential fitness of the variants, can arise in such protocells. In models of growing–dividing protocells that contain ACSs, daughter protocells inherit the composition of the mother, and this transmission of compositional information is the mechanism of heredity [11,26,27] instead of template replication of an information-carrying molecule. The interesting property of ‘synchronization’ has been shown to arise fairly generically in these models [19,28], whereby the composition of the protocell at successive divisions remains the same, giving the lineage of protocells a stable compositional identity. As a source of variation or ‘mutation’ (needed for evolution in protocells that do not have a genome), models have considered stochastic chemical fluctuations due to the chance occurrence of rare reactions which are enhanced in small volumes [9,11,21,29,30,31]. Other sources of variation considered are changes in the environment (e.g., addition or removal of molecular species from the food set) [27,31] and the exchange of molecules between protocells [21]. A large network containing multiple ACSs [32,33] can have multiple attractors in which different ACSs dominate. Transitions between such attractors initiated by stochastic fluctuations were proposed as a mechanism for the evolution of autocatalytic sets in a fixed volume [9]. It has been suggested [11] that this mechanism coupled with compartment growth could give rise to the differential fitness of protocells in different attractor states.

However, no model exists for which it has been shown that in a constant environment (1) the differential growth rates of the compartment in different attractors arise endogenously from the chemical dynamics (differential fitness), and (2) spontaneous stochastic fluctuations in a protocell lead to a change in the relative populations of the protocells in the different attractors (variation and selection). Therefore, a crisp and convincing theoretical demonstration of the Darwinian evolution of a population of ACS-containing protocells remains an unfinished task [13]. In this paper, we present a new model which explicitly demonstrates the evolution of a population of such protocells in the Darwinian sense (albeit only one step of evolution due to the simplicity of the model). Our work makes use of an interesting feature of certain autocatalytic network topologies: the presence of multistability in the dynamics [34,35,36,37,38]. Our protocell has just two stable states, one in which no ACS is present (inactive state) and the other in which it is (active state). The protocell has a higher growth rate in the active state compared to the inactive state. The variation in the protocell is just the spontaneous transition from the inactive to the active state due to a chemical fluctuation in a small volume, without any change in environment. The evolution exhibited is the establishment, growth, and dominance of the active protocells in a population of protocells. The simplicity of the model allows us to quantify the conditions under which this ‘natural selection’ can take place, in terms of the various dynamically generated timescales of the model. In future work, we hope to generalize this to multiple evolutionary steps of increasing complexity.

## 2. The Model

**Figure 1:**
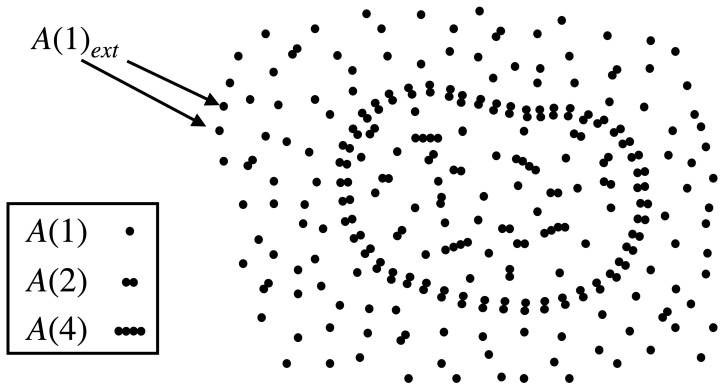
An illustration of a protocell inside an aqueous medium buffered with monomeric food molecules, $A{\left(1\right)}_{ext}$. The protocell membrane is composed of dimer molecules A(2).

The protocell consists of three molecular species: a monomer A(1) (food molecule), a dimer A(2) (assumed to be the enclosure-forming molecule), and a tetramer A(4) (catalyst); see Figure 1. The population of A(i)(i=1,2,4) in the protocell is denoted as $X_{i}$; $x_{i}\equiv X_{i}/V$ is its concentration, where *V* is the volume of the protocell. The set of reactions these molecules can undergo are:

$$\begin{array}{ccc} \mathrm{Transport}:\; & A{\left(1\right)}_{ext} & \overset{\alpha X_{2}}{⟶}A\left(1\right)\\ R1\left(\mathrm{uncatalyzed}\right):\; & 2A(1)\; & \overset{k_{F}}{\underset{k_{R}}{⇌}}\;\;A\left(2\right)\\ R1\left(\mathrm{catalyzed}\right):\; & 2A(1)+A(4)\; & \overset{\kappa k_{F}}{\underset{\kappa k_{R}}{⇌}}\;\;A\left(2\right)+A\left(4\right)\\ R2\left(\mathrm{uncatalyzed}\right):\; & 2A(2)\; & \overset{k_{F}}{\underset{k_{R}}{⇌}}\;\;A\left(4\right)\\ R2\left(\mathrm{catalyzed}\right):\; & 2A(2)+A(4)\; & \overset{\kappa k_{F}}{\underset{\kappa k_{R}}{⇌}}\;\;A\left(4\right)+A\left(4\right)\\ \mathrm{Degradation}:\; & A\left(2\right)\overset{\phi }{⟶}Ø, & \;\;A\left(4\right)\overset{\phi }{⟶}Ø. \end{array}$$

$A{\left(1\right)}_{ext}$ denotes the monomer species outside the cell; its concentration is assumed to be constant. The membrane formed by the dimers is permeable only to monomers; the rate at which monomers come in is proportional to the number of dimers, α being the proportionality constant. Two monomers can spontaneously ligate to form a dimer and two dimers to form a tetramer, both with the same rate constant $k_{F}$. The reverse (dissociation) reactions have a spontaneous rate constant $k_{R}$. These ligation–dissociation reactions are also catalyzed by the tetramer, whose ‘catalytic efficiency’ is denoted κ (this effectively means that the catalyzed reaction rate is $\kappa x_{4}$ times the spontaneous rate). The dimer and tetramer are assumed to degrade with rate constant ϕ into a waste product that quickly diffuses out of the protocell. Note that the catalyzed reactions R1 and R2 together with the transport reaction form an ACS starting from the food set $A{\left(1\right)}_{ext}$.

In this model, the dimer does double duty as both the enclosure-forming molecule as well as a reactant for catalyst production. In the equations below, we do not introduce separate population variables for the two roles. This is purely for simplicity and is not a crucial assumption. In the Supplementary Material, Section 1, we show that in a model with two monomer species in which these two functions are performed by distinct molecules, similar results arise.

Using mass action kinetics, the deterministic rate equations of the model are given by

(1) $$\begin{array}{cc} \frac{dx_{1}}{dt}= & \alpha x_{2}-2\left(k_{F}^{\prime }x_{1}^{2}-k_{R}^{\prime }x_{2}\right)-\frac{\dot{V}}{V}x_{1},\\ \frac{dx_{2}}{dt}= & k_{F}^{\prime }x_{1}^{2}-k_{R}^{\prime }x_{2} \end{array}$$

(2) $$\begin{array}{c} -2\left(k_{F}^{\prime }x_{2}^{2}-k_{R}^{\prime }x_{4}\right)-\left(\phi +\frac{\dot{V}}{V}\right)x_{2}, \end{array}$$

(3) $$\begin{array}{cc} \frac{dx_{4}}{dt}= & \left(k_{F}^{\prime }x_{2}^{2}-k_{R}^{\prime }x_{4}\right)-\left(\phi +\frac{\dot{V}}{V}\right)x_{4}, \end{array}$$

(4) $$\begin{array}{cc} k_{F}^{\prime }\equiv & k_{F}\left(1+\kappa x_{4}\right),\;k_{R}^{\prime }\equiv k_{R}\left(1+\kappa x_{4}\right). \end{array}$$

The $\dot{V}/V$ terms represent dilution in an expanding volume. Note that when *V* is not constant, Equations (1)–(3) do not specify the dynamics completely unless the growth rate $\dot{V}/V$ is specified. Since here we want an endogenous growth rate, we do not specify $\dot{V}/V$ exogenously. Instead, we write the model in terms of the populations, and assume a certain functional form for *V* in terms of the populations. In terms of $X_{i}$, the above equations reduce to

(5) $$\begin{array}{cc} \frac{dX_{1}}{dt}= & \alpha X_{2}-2\left(\frac{k_{F}X_{1}^{2}}{V}-k_{R}X_{2}\right)\left(1+\kappa \frac{X_{4}}{V}\right),\\ \frac{dX_{2}}{dt}= & \left(\frac{k_{F}X_{1}^{2}}{V}-k_{R}X_{2}\right)\left(1+\kappa \frac{X_{4}}{V}\right) \end{array}$$

(6) $$-2\left(\frac{k_{F}X_{2}^{2}}{V}-k_{R}X_{4}\right)\left(1+\kappa \frac{X_{4}}{V}\right)-\phi X_{2},$$

(7) $$\frac{dX_{4}}{dt}=\left(\frac{k_{F}X_{2}^{2}}{V}-k_{R}X_{4}\right)\left(1+\kappa \frac{X_{4}}{V}\right)-\phi X_{4}.$$

For simplicity we take *V* to be a linear function of the populations $X=(X_{1},X_{2},X_{4})$:

(8) $$V\left(X\right)=v\left(X_{1}+2X_{2}+4X_{4}\right),$$

where *v* is a constant. This choice gives the protocell a constant mass density (as observed in bacterial cells [39]) since *V* is proportional to the mass of the protocell. This choice is not essential; we have tried other linear functions $V=v_{1}X_{1}+v_{2}X_{2}+v_{4}X_{4}$ ($v_{i}$ constant), including $V=v(X_{1}+X_{2}+X_{4})$. The quantitative results depend on the values of $v_{i}$ but the qualitative features presented below hold for all the cases considered. We have also considered other versions of the model with the transport term $\alpha X_{2}$ in Equation (5) modified to a gradient term $\alpha X_{2}\left(x_{1,\mathrm{ext}}-x_{1}\right)$ (where $x_{1,\mathrm{ext}}$ is the constant concentration of $A{\left(1\right)}_{ext}$), certain other autocatalytic reaction topologies, etc. (see Supplementary Material, Section 1). The qualitative conclusions seem to be robust to these choices. Without loss of generality, the constants $k_{R}$ and *v* are set to unity by rescaling $t\to k_{R}t$, $\alpha \to \alpha /k_{R}$, $\phi \to \phi /k_{R}$, $k_{F}\to k_{F}/\left(k_{R}v\right)$, and κ→κ/v, which makes time and the other parameters dimensionless.

The definition of V(X) and the values of the rescaled parameters $k_{F},\phi ,\alpha$, and κ completely define Equations (5)–(7), and one can solve for X(t) given any initial condition. In a particular trajectory, *V* may increase or decrease. Protocells larger than a characteristic size may become floppy or unstable and spontaneously break up into smaller entities. We assume that if *V* increases to a critical value $V_{c}$, the cell divides into two identical daughters, each containing half of the three chemicals of the mother protocell at division. The dynamics of a daughter after division is again governed by Equations (5)–(8). This division rule and Equations (5)–(8) together completely define the model at the deterministic level.

The dynamics of the ACS consisting of the catalyzed reactions R1 and R2 in a fixed-size container but with buffered A(1) as the food set is given by Equations (6) and (7), with *V* and $X_{1}$ being constant. This was studied in [37] at the deterministic level, where a bistability was observed, and in [38] at the stochastic level, where transitions between the attractors were observed. The present model, by adding Equations (5) and (8) and the division rule, embeds the ACS in a growing–dividing protocell instead of a fixed-volume container. It shares the bistability of the fixed-volume version, but also possesses qualitatively new properties. These properties (considered along with stochastic dynamics) enable a population of such protocells to mimic (one step of) Darwinian evolution, as will be discussed below.

## 3. Results

### 3.1. Deterministic Dynamics: Bistability with Two Distinct Growth Rates

Since *V* is a linear function of the populations, $\dot{V}/V$ can be expressed in terms of the concentrations. Differentiating Equation (8) with respect to *t* and using Equations (5)–(7), it follows that

(9) $$\mu \equiv \frac{\dot{V}}{V}=\alpha x_{2}-\phi \left(2x_{2}+4x_{4}\right).$$

Equation (9) expresses the instantaneous growth rate of the protocell in terms of its chemical composition, a feature that is missing from previous protocell models.

**Figure 2:**
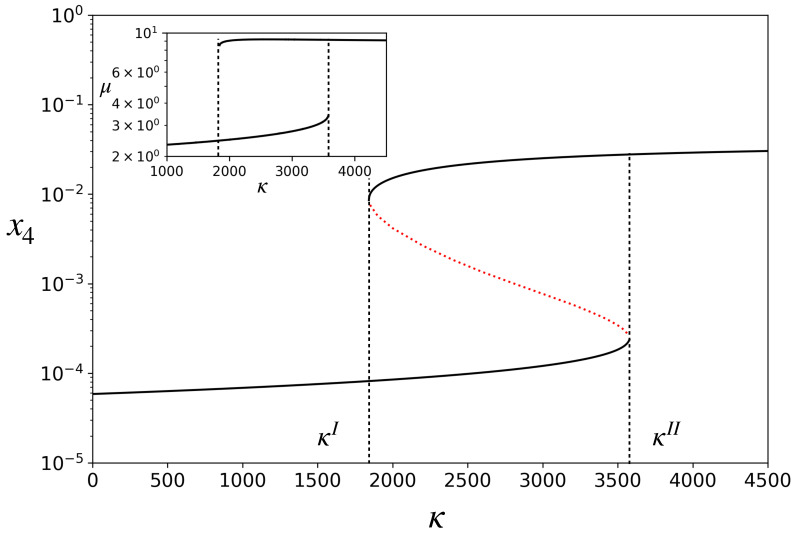
Bifurcation diagram for the model: steady state concentration, $x_{4}$, of the catalyst versus catalytic efficiency, κ. The region between $\kappa^{I}\left(=1840\right)$ and $\kappa^{II}\left(=3580\right)$ is the region having three fixed points, two of which are stable (solid black curves) and one is unstable (red dotted curve). **Inset**: Growth rate, μ, of the protocell, versus κ. Parameters: hereafter, $k_{R}$ and *v* have been set to unity without loss of generality after non-dimensionalizing the model. $k_{F}=1$, ϕ=20, α=100.

**Figure 3:**
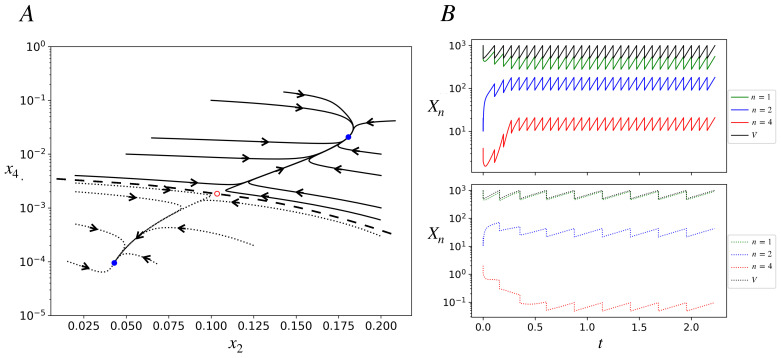
Deterministic trajectories in the bistable region of the model. κ=2400; other parameters are as in Figure 2. The time, *t*, is in dimensionless units in this figure and subsequent figures (effectively *t* is measured in units of 1/$k_{R}$). (**A**) Phase portrait projected onto the $x_{2}-x_{4}$ plane. Several trajectories starting with different initial conditions are shown; they reach one of two stable fixed points denoted by blue closed dots. All the solid curve trajectories end at the stable fixed point on the top right (ACS-active), while all the dotted trajectories end at the stable fixed point on the bottom left of the plot (ACS-inactive). The red open dot represents an unstable fixed point. The dashed curve is a schematic of the basin boundary between the two stable fixed-point attractors. (**B**) Deterministic trajectories of populations (in log scale) of species A(1), A(2), and A(4) and the protocell volume as functions of time for two initial conditions. $V_{c}=1000$. Initial conditions: IC1 (lower panel; dotted curves): $X_{1}=952,X_{2}=20,X_{4}=2$. IC2 (upper panel; solid curves): $X_{1}=944,X_{2}=20,X_{4}=4$. Protocell starting with IC1 ends up in the *inactive state*, in which the population of the catalyst A(4) is less than one, as seen in dotted red curve in the lower panel. Protocell starting with IC2 ends up in the *active state*, in which the population of the catalyst is high (approximately between 10 and 20). The interdivision times in the inactive and active states are, respectively, $\tau_{1}=0.269$, $\tau_{2}=0.075$.

When Equation (9) is substituted into Equations (1)–(3), the concentration dynamics also becomes completely defined. It has fixed points. Figure 2 shows a bifurcation diagram in which the fixed-point concentration of A(4) is plotted by varying the parameter κ. The model exhibits bistability for $\kappa^{I}<\kappa <\kappa^{II}$. Note that the catalyst concentration $x_{4}$ in the upper stable branch is two orders of magnitude higher than in the lower stable branch. On the lower branch, the rates of catalyzed reactions are smaller than the corresponding spontaneous reactions, while on the upper branch they are much higher. We, therefore, refer to the upper branch as one in which the ACS is *active* and the lower branch as ACS-*inactive*. Depending on the initial condition, for a given κ in the bistable region, the dynamics will settle into either of the two stable attractors, as shown in Figure 3A for one such κ. For $\kappa <\kappa^{I}$, there is only one attractor (the inactive one), and for $\kappa >\kappa^{II}$, there is also only one attractor (the active one).

For each fixed-point attractor, the right-hand side of Equation (9) is constant. Hence, in the attractor, *V* grows exponentially, $V\left(t\right)=V\left(0\right)e^{\mu t}$ with constant μ. In other words, the protocell has a characteristic growth rate in each attractor given by the expression in Equation (9). This is shown in the inset of Figure 2. Hence, in the bistable region, the protocell can grow with two distinct growth rates depending upon which attractor it is in. The growth rate is many times higher in the active state than in the inactive one.

Once the concentrations have reached their fixed-point attractor, (9) implies that *V* grows exponentially, and Equation (8) then implies that each chemical population must also grow exponentially with the *same* rate, μ. (Only if all populations grow at the same rate as *V* will their concentrations be constant). Thus, in each attractor we have $X_{i}\left(t\right)=X_{i}\left(0\right)e^{\mu t}$. In other words, the protocell naturally exhibits *balanced growth* in each attractor (growth with ratios of all populations constant [40]). Exponentially growing trajectories in a nonlinear system and this remarkable emergent coordination between the chemicals without any explicit regulatory mechanism are a consequence of (a) the fact that the right-hand sides of Equations (5)–(7) are homogeneous degree-one functions of the populations (if all three populations are simultaneously scaled by a factor β, $X_{i}\to \beta X_{i}$, then the right-hand sides of Equations (5)–(7) also scale by the same factor β), and (b) that the ACS structure couples all chemicals to each other. This is discussed in detail in Ref. [41] in the context of models of bacterial physiology.

Figure 3B shows, for a protocell, the trajectories of its chemical populations and volume as functions of time for two very close initial conditions (defined by the population of species A(1), A(2), and A(4)) that lie in different attractor basins. They converge to different attractors: ACS-active (upper panel) and inactive (lower panel). After a protocell divides we track one of its daughters. The attractor is a fixed point for concentrations (Figure 3A) but a limit cycle for populations and the volume (Figure 3B). The growth phase of the limit cycle has the same constant slope for all populations in a given attractor, signifying exponential growth with the same growth rate for all chemicals in the attractor. The slope is larger (and interdivision time shorter) for the active attractor. At division, since populations and the volume both halve, concentrations do not see any discontinuity.

The existence of bistability is robust in the parameter space. It may be noted that a nonzero degradation rate ϕ of the dimer and tetramer is essential for bistability (as also found in the model studied in Ref. [37]). A degradation term $\phi^{\prime }x_{1}$ for the monomer can also be introduced in Equation (1); however, it is found that $\phi^{\prime }$ must be sufficiently smaller than ϕ for bistability to exist.

### 3.2. Stochastic Dynamics of a Single Protocell: Transitions between States of Different Growth Rates

We now consider the protocell under the stochastic chemical dynamics framework. The chemical populations are now non-negative integers and each unidirectional reaction occurs with a probability that depends on the populations of the reactants and the values of the rate constants. We simulate the stochastic chemical dynamics of the protocell using the Gillespie algorithm [42]. The reaction probabilities are listed in Appendix A. Whenever a reaction occurs, the populations of its reactants and products are updated. For large populations, when fluctuations are ignored, the above-mentioned probabilities lead to the deterministic Equations (1)–(3) or (5)–(7). In using the Gillespie algorithm for expanding volumes, the rate of increase in the volume needs to be taken into account [43,44]. In the present work, since volume is treated as a function of populations (8), we assume that it is instantaneously updated when the populations are.

**Figure 4:**
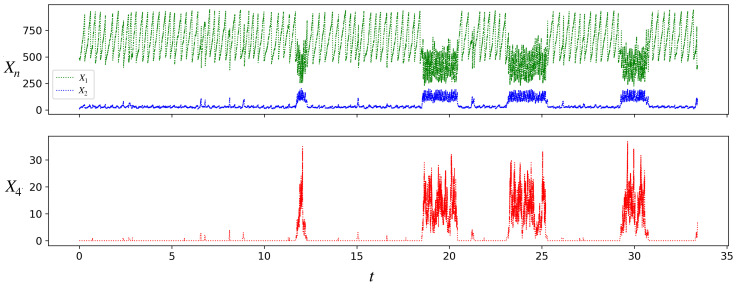
Stochastic simulation of the populations of species A(1), A(2), and A(4) for a single protocell lineage in the model. Parameter values are as in Figure 3, $V_{c}=1000$. Initial condition: $X_{1}=480,\;X_{2}=10,\;X_{4}=0$. Note the transitions of the protocell between the inactive and active states. From a long such simulation we find that the average interdivision times in the inactive and active states are, respectively, $\langle \tau_{1}\rangle =0.295$, and $\langle \tau_{2}\rangle =0.077$, while the average residence times in the two states are $\langle T_{1}\rangle =3.413$, and $\langle T_{2}\rangle =1.916$.

Figure 4 shows a simulation run of the stochastic chemical dynamics of a single growing and dividing protocell. At the volume threshold $V_{c}$, when the protocell divides into two daughter protocells, we implement partitioning stochasticity, namely, each molecule in the mother is given equal probability of going into either daughter. In Figure 4, at each division we randomly discard one of the two daughters and choose one for further tracking, in order to display a single-cell trajectory over several divisions (effectively it is the trajectory of a single lineage of protocells).

**Figure A1:**
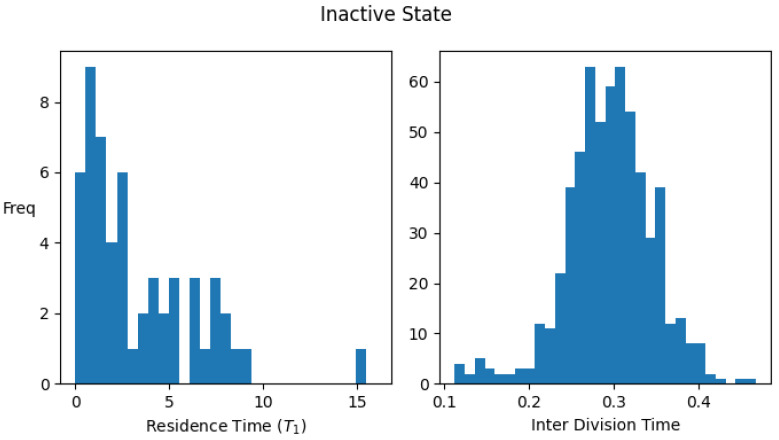

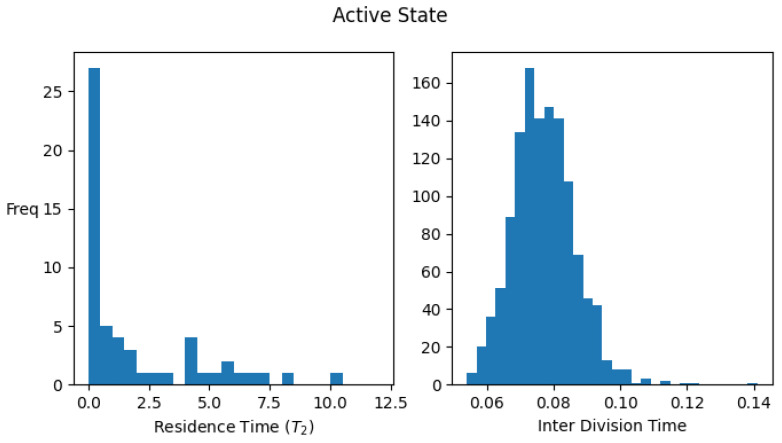
Distribution of residence times (time spent) and interdivision time in active and inactive states. Data were collected by simulating a single lineage of growing and dividing protocells over 2000 division cycles. Parameter values: $\kappa =2400,\;k_{F}=1,\;\phi =20,\;\alpha =100$. The average values of the interdivision times are $\langle \tau_{1}\rangle =0.2947$ and $\langle \tau_{2}\rangle =0.0772$, while the average residence times in the two states are $\langle T_{1}\rangle =3.413$ and $\langle T_{2}\rangle =1.916$.

Starting from the initial condition shown, where the protocell is composed of only A(1) and A(2), the protocell initially grows and divides in the inactive state. The first A(4) molecule is produced by the chance occurrence of the uncatalyzed reaction 2. Production of a sufficient number of A(4) molecules triggers a transition to the active state, where the population of A(4) is significantly larger than in the inactive state. As in Figure 3 for the deterministic case, so also in Figure 4 it can be seen that the protocell in the active state grows and divides faster than in the inactive state. However, unlike the deterministic case, we also see transitions between the inactive and active states. These transitions occur because for a small protocell (500≤V≤1000 for the protocell in Figure 4), chance production or depletion of a few molecules of A(4) is enough to push its concentration into the basin of the other attractor. Note that in Figure 4, the protocell lineage spends more time in the inactive state than the active. The residence times of a protocell lineage in the two attractors ($T_{1},T_{2}$) have distributions (see Figure A1 in Appendix B) that vary with the parameters.

Note that typically a daughter naturally inherits the state of the mother protocell: since the two daughters have roughly half the number of molecules of each type as the mother, and hence also half the volume, they have the same concentration of each chemical as the mother. Partitioning stochasticity occasionally results in a daughter losing the mother’s state.

### 3.3. Protocell Population Dynamics: Dominance of the Autocatalytic State

**Figure 5:**
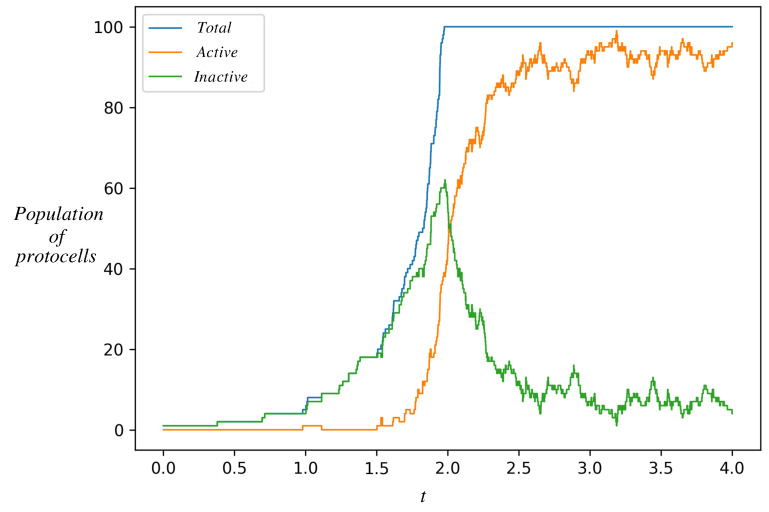
Time evolution of a population of protocells starting from a single protocell in the inactive state. Shown is the number of protocells in the inactive state (green), active state (orange), and their sum (blue). As inactive protocells grow and divide, their population increases. The orange curve departs from zero when one of the inactive protocells makes a stochastic transition to the active state. The two populations have different growth rates. After the total population reaches an externally imposed ceiling *K* (=100 in this figure), upon each cell division a randomly chosen protocell is removed from the population. The population eventually settles down in a stochastic steady state dominated by the active protocells. This is the natural selection of an autocatalytic state. Parameter values are as in Figure 4, K=100.

Figure 5 shows the time evolution of a population of such protocells. At t=0 we start from a single protocell in the inactive state, whose dynamics was shown in Figure 4. However, in this simulation, when a protocell divides, instead of discarding a daughter, we keep it in the simulation until the total population of protocells reaches an externally imposed ceiling *K*. After the total number of cells reaches *K*, the total population is kept constant. This is achieved by removing one randomly chosen protocell from among the K+1 protocells whenever any protocell divides. Each protocell in the population is independently simulated by the single-cell stochastic dynamics (Gillespie algorithm). Figure 5 tracks only the number of protocells in each state (active or inactive) as a function of time.

The number of protocells in the inactive state increases whenever one of them divides. Eventually, one of them makes a stochastic transition to the active state, whereupon the number of active protocells jumps from zero to one. Active protocells also make stochastic transitions to the non-active state on a certain timescale. However, since active protocells divide faster (as seen in Figure 4), their number grows faster and their population catches up and overtakes the inactive population in Figure 5. Eventually, the active protocells dominate the population.

The curves in Figure 5 represent the net result of stochastic transitions and proliferation by division. The fraction of protocells in each state is expected to reach a stochastic steady state (see below) that represents a balance between proliferation and transition. In the simulations, we find that the fraction of inactive cells declines when the total population hits *K* (see Figure 5). It eventually reaches its steady state fraction. A decline is seen at the time that the total population hits *K*, because in this simulation at that time the fraction of inactive cells is higher than its steady state fraction (this is a consequence of the initial condition, the fact that at t=0 we started from a single protocell in the *inactive* state). In Supplementary Material, Section 2, a similar qualitative behavior can be seen for other values of κ within the bistability region.

An approximate (mean field) model of the protocell population dynamics (valid for large populations) with no ceiling (K→∞) is the following:

(10) $$\frac{dn_{1}}{dt}=\mu_{1}n_{1}-\lambda_{1}n_{1}+\lambda_{2}n_{2},$$

(11) $$\frac{dn_{2}}{dt}=\mu_{2}n_{2}-\lambda_{2}n_{2}+\lambda_{1}n_{1},$$

where $n_{1}\left(n_{2}\right)$ is the population of protocells in the inactive (active) state, $\mu_{1}=\frac{\ln2}{\langle \tau_{1}\rangle }$ and $\mu_{2}=\frac{\ln2}{\langle \tau_{2}\rangle }$ are the average growth rates of the protocell in the inactive and active states, respectively, and $\lambda_{1}=\frac{1}{\langle T_{1}\rangle }$ and $\lambda_{2}=\frac{1}{\langle T_{2}\rangle }$ are the transition rates, respectively, from the inactive to active and active to inactive states. This is a linear dynamical system $\frac{dn}{dt}=An$, where $n={\left(n_{1}\;\;n_{2}\right)}^{T}$ is the column vector of protocell populations, and

(12) $$A=\left(\begin{array}{cc} \mu_{1}-\lambda_{1} & \lambda_{2}\\ \lambda_{1} & \mu_{2}-\lambda_{2} \end{array}\right).$$

Equations (10) and (11) for the populations of inactive and active protocells are identical to the model used to describe the populations of persister and normal cells of bacteria [45].

The steady state fraction *f* of active protocells in the population $f\equiv n_{2}/\left(n_{1}+n_{2}\right)$ can be computed from the eigenvector of *A* corresponding to its largest eigenvalue, $e_{1}$. The result is

(13) $$f=\frac{\lambda_{1}}{e_{1}+\lambda_{1}+\lambda_{2}-\mu_{2}},$$

where $e_{1}=\frac{1}{2}\left[\mathrm{tr}\left(A\right)+\sqrt{{\left(\mathrm{tr}\left(A\right)\right)}^{2}-4\det\left(A\right)}\right]$, $\mathrm{tr}\left(A\right)=\mu_{1}-\lambda_{1}+\mu_{2}-\lambda_{2}$, and $\det\left(A\right)=\left(\mu_{1}-\lambda_{1}\right)\left(\mu_{2}-\lambda_{2}\right)-\lambda_{1}\lambda_{2}$. A calculation of *f* for a finite but large-ceiling *K* is given in Appendix C.1 and yields the same answer as (13), independent of *K*.

Using the averages given in the caption of Figure 4 to determine the components of *A*, this calculation yields f=0.925±0.014 (mean ± standard error), with the error arising from the finite sample estimation of the averages. This agrees with the fraction found (over long times) in the stochastic steady state of the simulation of Figure 5, namely, 0.937±0.019 (mean ± standard deviation). The Supplementary Material, Section 3, shows the agreement between the simulations and the mean-field model at other values of κ.

Note in Figure 5, that even though the active protocells have a higher growth rate than the inactive, a finite fraction of the inactive still survives in the steady state. This is because of the nonzero transition probability $\lambda_{2}$ from the active to the inactive state. If $\lambda_{2}$ had been zero, the eigenvector of *A* corresponding to its largest eigenvalue would have been ${\left(0\;\;1\right)}^{T}$, implying that the inactive state is extinct in the steady state. When $\lambda_{2}\neq 0$, one can show (see Appendix C.2) that if

(14) $$\lambda_{2}\ll \mu_{2}-\mu_{1}+\lambda_{1},$$

then $f≃1-\frac{\lambda_{2}}{\mu_{2}-\mu_{1}+\lambda_{1}}$ is close to unity. The quantity $1/(\mu_{2}-\mu_{1}+\lambda_{1})$ defines a certain timescale of the single protocell dynamics. The above condition means that if the average lifetime $\langle T_{2}\rangle$ ($=\frac{1}{\lambda_{2}}$) of the active state is much larger than this timescale, ACS-active protocells will come to dominate the population. Another way of writing this condition is $\mu_{2}\langle T_{2}\rangle -\mu_{1}\langle T_{2}\rangle +\frac{\langle T_{2}\rangle }{\langle T_{1}\rangle }\gg 1$. Therefore, a sufficient condition for active protocells to dominate is that the active protocell divides many times in its typical lifetime ($\mu_{2}\langle T_{2}\rangle \gg 1$) *and* grows much faster than the inactive protocell ($\mu_{2}\gg \mu_{1}$).

Note also that a nonzero $\lambda_{1}$ is what ensures that even if we start with a zero population of active protocells, one active protocell will sooner or later be produced by chance, leading eventually to a fraction *f* of active protocells.

## 4. Discussion And Conclusions

In this work, we have constructed an example that shows (i) how autocatalytic sets of reactions inside protocells can spontaneously boost themselves into saliency and enhance the populations of their product molecules including catalysts, and (ii) how such protocells (where the ACS is active) can come to dominate in a population of protocells. Encasement within protocells serves two important functions. (i) The small size of a protocell allows a small number fluctuation of the catalyst molecules to take their concentration past the basin boundary of the attractor in which the ACS is inactive into the basin of the active attractor, thereby causing the protocell to transition from an inactive to active state. A large container would require a larger number fluctuation to achieve the same transition, which is more unlikely. (ii) Protocells in the active state grow at a faster rate than the inactive state, thereby eventually dominating in the population. The differential growth rate is a consequence of the fact that the protocell size depends upon its internal chemical populations, a possibility that is precluded when we discuss chemical dynamics in a fixed-size container. Therefore, in this example, protocells aid both the generation and the amplification of autocatalytic sets.

The differential growth rates of the two states are not posited exogenously, but arise endogenously within the model from the underlying chemical dynamics defined by Equations (5)–(8) (and their stochastic version). The additional assumption made is that upon reaching a critical size a protocell divides into two daughters that share its contents. This property can arise naturally due to some physical instability. Collectively these assumptions lead to the properties of heredity, heritable variation (the variation is heritable because once the fluctuation pushes it into a new basin of attraction a protocell typically descends into its new attractor in a short time), and differential fitness in a purely physico-chemical system. This leads to the dynamics of the two subpopulations of protocells shown in Figure 5, which is similar to that of natural selection. (A difference is that the slower-growing subpopulation never goes completely extinct, due to the nonzero probability of the transition of a faster-growing protocell into a slower-growing one).

The process of going from an initial state with no ACS to its establishment in a population of protocells, discussed here, might be considered the first step in the evolution of the ACS. One might wonder how the ACS would evolve further from there. It has been shown that chemistries containing ACSs exhibit multistability in fixed-size containers. In some of these chemistries, simpler ACSs involving small catalyst molecules are nested inside more complex ACSs having larger and more efficient catalyst molecules [37]. The multiple attractor states correspond to ACSs with progressively larger molecules and higher levels of complexity being active. It is possible that by embedding such chemistries within protocells, the mechanism discussed here could allow one to realize a punctuated evolutionary path through sequentially more complex ACS attractors to a state of high chemical complexity from an initial state that only contains small molecules and no ACS. This is a task for the future.

The specific artificial chemistry and protocell properties studied here are highly idealized ones. The object was to demonstrate a mechanism in principle. However, we believe the mechanism is quite general and it should be possible to demonstrate it in other models (e.g., [11,27]) provided multistability in a fixed environment and the emergence of distinct timescales, as discussed in the present work, can be established. We remark that although we have been primarily thinking of protocells as vesicles (motivated by similar models of bacterial physiology), some of our methods might also be useful in the context of micelles. Recently, Kahana et al [31] presented a model of the stochastic dynamics of lipid micelles which had multiple attractors corresponding to distinct composomes. It would be interesting to compare the growth rates of micelles in different attractors as well as the transition rates between the attractors in their model.

We note that there have been independent experimental developments in constructing bistable autocatalytic chemistries [46] and self-replicating protocells [22]. It is also established that small peptides exhibit catalytic properties [47] and they can be encapsulated within protocells to promote protocellular growth [48]. A recent paper also shows the coupling of a simple autocatalytic reaction with the compartment growth and division [49]. A synthesis of these approaches might result in the experimental realization of the mechanism described in the present work.

We have considered dynamics at three levels. One is the chemical dynamics of molecules within a single protocell. This depends upon molecular parameters such as rate constants, the efficiency of the catalyst molecule, etc. From this, we extracted effective parameters at the second level: that of a single protocell (growth rates of the two protocell states, residence times, etc.). These were then used to derive the dynamics at the third level, consisting of the population of protocells. This enabled an understanding of the conditions under which active protocells would dominate. Such an approach might be useful in other settings, for example, in understanding certain aspects of bacterial ecology from molecular models of single bacterial cells.

To conclude, we have shown that in theory, under suitable assumptions, a compartment containing an autocatalytic set of small molecules can self-replicate, produce heritable variation, and evolve in the Darwinian sense to a state of higher complexity. Notably, the model also tells us the conditions required on various timescales for this to happen. This may help in further exploring, both theoretically and experimentally, the evolutionary capabilities of simple molecular systems that precede the origin of life.

## Data Availability

The data relevant for this work are contained within the article or the supplementary material.

## Supplementary Materials

The following supporting information can be downloaded at: https://www.mdpi.com/article/10.3390/life13122327/s1, Figure S1: Bifurcation diagram for the 5-chemical-species model; Figure S2: Stochastic simulation of population of species *A*(1), *B*(1), *A*(2), *B*(2) and *A*(4) for the model using the Gillespie algorithm; Figure S3: Time evolution of a population of protocells in the 5-chemical-species model starting from a single protocell in the inactive state; Figure S4: Simulations of the model at κ = 2000; Figure S5: Simulations of the model at κ = 3400; Figure S6: Fraction f of ‘ACS active’ protocells in the stochastic steady state of the protocell population dynamics versus κ, obtained from simulation (black hollow circles) and the mean field model (red solid dots).

## Abbreviations

The following abbreviations are used in this manuscript:

ACS	Autocatalytic set

## Appendix A. Reaction Probabilities Used in Gillespie Algorithm

**Table A1:** List of unidirectional reactions in the model and their reaction probabilities per unit time. *V* appearing in the table is given by the right-hand side of Equation (9) in the main paper text. Deterministic rates of reactions in the last column are given as in Equations (5)–(7) of the main paper.

Reaction	Reaction Type	Reaction Probability per Unit Time	Deterministic Rate of Reaction
$A{\left(1\right)}_{ext}+A\left(2\right)\overset{\alpha X_{2}}{⟶}A\left(1\right)+A\left(2\right)$	transport	$\alpha X_{2}$	$\alpha X_{2}$
$A\left(1\right)+A\left(1\right)\overset{k_{F}}{⟶}A\left(2\right)$	spontaneous	$k_{F}X_{1}\left(X_{1}-1\right)V^{-1}$	$k_{F}X_{1}^{2}V^{-1}$
$A\left(1\right)+A\left(1\right)+A\left(4\right)\overset{\kappa k_{F}}{⟶}A\left(2\right)+A\left(4\right)$	catalyzed	$\kappa k_{F}X_{4}V^{-1}X_{1}\left(X_{1}-1\right)V^{-1}$	$\kappa k_{F}X_{4}V^{-1}X_{1}^{2}V^{-1}$
$A\left(2\right)+A\left(2\right)\overset{k_{F}}{⟶}A\left(4\right)$	spontaneous	$k_{F}X_{2}\left(X_{2}-1\right)V^{-1}$	$k_{F}X_{2}^{2}$
$A\left(2\right)+A\left(2\right)+A\left(4\right)\overset{\kappa k_{F}}{⟶}A\left(4\right)+A\left(4\right)$	catalyzed	$\kappa k_{F}X_{4}V^{-1}X_{2}\left(X_{2}-1\right)V^{-1}$	$\kappa k_{F}X_{4}V^{-1}X_{2}^{2}$
$A\left(2\right)\overset{k_{R}}{⟶}A\left(1\right)+A\left(1\right)$	spontaneous	$k_{R}X_{2}$	$k_{R}X_{2}$
$A\left(2\right)+A\left(4\right)\overset{\kappa k_{R}}{⟶}A\left(1\right)+A\left(1\right)+A\left(4\right)$	catalyzed	$\kappa k_{R}X_{4}V^{-1}X_{2}$	$\kappa k_{R}X_{4}V^{-1}X_{2}$
$A\left(4\right)\overset{k_{R}}{⟶}A\left(2\right)+A\left(2\right)$	spontaneous	$k_{R}X_{4}$	$k_{R}X_{4}$
$A\left(4\right)+A\left(4\right)\overset{\kappa k_{R}}{⟶}A\left(2\right)+A\left(2\right)+A\left(4\right)$	catalyzed	$\kappa k_{R}X_{4}V^{-1}\left(X_{4}-1\right)$	$\kappa k_{R}X_{4}^{2}V^{-1}$
$A\left(2\right)\overset{\phi }{⟶}Ø$	degradation	$\phi X_{2}$	$\phi X_{2}$
$A\left(4\right)\overset{\phi }{⟶}Ø$	degradation	$\phi X_{4}$	$\phi X_{4}$

## Appendix B. Single-Cell Residence Time and Interdivision Time Distributions for Active/Inactive States of the Protocell

### Appendix B.1. Definition of an Active/Inactive State of a Protocell

In order to obtain residence times and interdivision times in the active and inactive states of the protocell, an inference has to be made from the intracellular populations as to whether the protocell is in the active state or inactive state. The protocell was defined to be in the active (inactive) state if its concentration profile was in the basin of attraction of the active (inactive) attractor. The two basins are separated by a basin boundary (as shown by the black dashed curve in Figure 3A of the main paper). One might choose an alternative criterion based on ‘closeness’ to the attractor state, but for the purposes of the present work, the above definition is useful. In practice, for simplicity in the present work, the concentration of the catalyst molecule ($x_{4}$) at the unstable fixed point (through which the basin boundary passes) was taken to be the threshold value for determining the state of the protocell. If $x_{4}$ was above this value, the cell was labeled as active, otherwise it was labeled as inactive. This is an approximate implementation of the above definition. While the actual values of transition times would change when the above definition is implemented exactly, we do not expect our qualitative conclusions to depend significantly on this approximation.

### Appendix B.2. Definition of Residence Time and Interdivision Time

While tracking the trajectory of a single lineage of cells (as shown in Figure 4 of the main paper) the state of the protocell (1 if active; 0 if inactive) was determined for the mother protocell at every division along with the time of the division event. (For more details on data generation, see Section 4 of the Supplementary Material). A long such trajectory gave a sequence of division times and a corresponding sequence of ones and zeros. A contiguous subsequence consisting of only ones bordered by zeros (only zeros bordered by ones) at both ends of the subsequence was declared to be an instance of residence in the active state (inactive state). The duration of such a subsequence (equal to the difference between the ending and starting times of the subsequence as measured by the corresponding division times) was taken to be the lifetime of the state. Within an active or inactive subsequence, the difference between two consecutive division times was taken to be an instance of an interdivision time in that state.

Figure A1 shows the histograms for the residence times and interdivision times in the active and inactive states using the above definitions, for one set of parameter values.

## Appendix C. The Steady State Fraction of ACS-Active Protocells (*f*) in the Protocell Population

An expression was derived for the asymptotic fraction of active protocells in the protocell population dynamics (Equation (13) of the main paper). The derivation used mean-field equations for the populations of the active and inactive protocells and assumed indefinite growth of the two populations. Here, we show that the same expression follows if we truncate the total population of protocells at a large-ceiling *K*. We analyze the conditions under which this fraction is close to unity. We also present numerical evidence that the fraction so obtained agrees with the actual stochastic simulations of protocell population dynamics at different values of κ.

### Appendix C.1. Calculation of f for a System with Finite-Ceiling K on the Total Population

In our stochastic simulations of protocell population dynamics, the total number of protocells increases until it reaches the ceiling *K*. After that, it becomes constant because whenever a protocell divides, one protocell chosen at random is removed from the population. Consider the dynamics of $n_{1}$ and $n_{2}$ (populations of the inactive and active protocells, respectively) after the total population $n_{1}+n_{2}$ has reached this constant value *K*. If *K* is sufficiently large, we can use the same equations as before (namely, Equations (10) and (11) of the main text) modified by the addition of a death term on the right-hand side. In other words,

(A1) $${\dot{n}}_{1}=\mu_{1}n_{1}-\lambda_{1}n_{1}+\lambda_{2}n_{2}-\beta n_{1}$$

(A2) $${\dot{n}}_{2}=\mu_{2}n_{2}-\lambda_{2}n_{2}+\lambda_{1}n_{1}-\beta n_{2},$$

where the last term in both equations accounts for the removal of active or inactive protocells in proportion to their existing population (the average effect of the random removal of a protocell from the population). β is chosen so that the total population is constant, i.e., $n_{1}+n_{2}=K$. Then, using ${\dot{n}}_{1}+{\dot{n}}_{2}=0$, we obtain

(A3) $$\beta =\frac{\mu_{1}n_{1}+\mu_{2}n_{2}}{n_{1}+n_{2}}=\frac{\mu_{1}n_{1}+\mu_{2}n_{2}}{K}.$$

Eliminating $n_{1}=K-n_{2}$ from the ${\dot{n}}_{2}$ equation, and setting ${\dot{n}}_{2}=0$ to obtain a fixed point, we obtain a quadratic equation for the fixed-point value of $n_{2}$:

$$\frac{\left(\mu_{2}-\mu_{1}\right)}{K}n_{2}^{2}-\left(\mu_{2}-\mu_{1}-\lambda_{2}-\lambda_{1}\right)n_{2}-\lambda_{1}K=0.$$

This has the solution

(A4) $$n_{2}=\frac{K}{2(\mu_{2}-\mu_{1})}\left[\left(\mu_{2}-\mu_{1}-\lambda_{2}-\lambda_{1}\right)\pm \sqrt{{\left(\mu_{2}-\mu_{1}-\lambda_{2}-\lambda_{1}\right)}^{2}+4\lambda_{1}\left(\mu_{2}-\mu_{1}\right)}\right].$$

When $\mu_{2}-\mu_{1}$ is positive (as is the case in our simulations), the positive root must be chosen to obtain a physical solution (non-negative value of $n_{2}$). This yields

(A5) $$f\equiv \frac{n_{2}}{K}=\frac{1}{2(\mu_{2}-\mu_{1})}\left[\left(\mu_{2}-\mu_{1}-\lambda_{2}-\lambda_{1}\right)+\sqrt{{\left(\mu_{2}-\mu_{1}-\lambda_{2}-\lambda_{1}\right)}^{2}+4\lambda_{1}\left(\mu_{2}-\mu_{1}\right)}\right].$$

The expression of *f* is independent of *K*. A bit of algebra shows that this expression is identical to that in Equation (13) of the main paper.

### Appendix C.2. Condition for Active Protocells to Dominate the Population

The above expression for *f* can be written as

(A6) $$\begin{array}{cc} f & =\frac{1}{2}\left[1-\frac{\lambda_{1}+\lambda_{2}}{\mu_{2}-\mu_{1}}+\sqrt{1+{\left(\frac{\lambda_{1}+\lambda_{2}}{\mu_{2}-\mu_{1}}\right)}^{2}+\frac{2(\lambda_{1}-\lambda_{2})}{\mu_{2}-\mu_{1}}}\right] \end{array}$$

(A7) $$\begin{array}{c} =\frac{1}{2}\left[1-s+\sqrt{1+s^{2}+2d}\right], \end{array}$$

where $s=\frac{\lambda_{1}+\lambda_{2}}{\mu_{2}-\mu_{1}}$ and $d=\frac{\lambda_{1}-\lambda_{2}}{\mu_{2}-\mu_{1}}$. This shows that *f* only depends upon the two dimensionless combinations *s* and *d* of the four parameters.

From the above expression, it immediately follows that f=1 when $\lambda_{2}=0$, as already mentioned in the main text. We can also ask: How small should $\lambda_{2}$ be for *f* to be close to unity? To see this it is useful to introduce the combinations $z=\frac{\lambda_{1}}{\mu_{2}-\mu_{1}}$ and $x=\frac{\lambda_{2}}{\mu_{2}-\mu_{1}}$. Then,

(A8) $$f=\frac{1}{2}\left[1-\left(z+x\right)+\sqrt{1+{\left(z+x\right)}^{2}+2\left(z-x\right)}\right]=\frac{1}{2}\left[1-\left(z+x\right)+\left(z+1\right)\sqrt{1+\frac{2\left(z-1\right)x+x^{2}}{{\left(z+1\right)}^{2}}}\right].$$

When $\frac{x}{z+1}\ll 1$, the second term inside the square root is much smaller than unity. Performing a Taylor expansion, we obtain $f≃1-\frac{x}{z+1}$ to leading order in $\frac{x}{z+1}$. This shows that the condition for the active protocells to dominate in the steady state of the population dynamics is

(A9) $$\frac{x}{z+1}=\frac{\lambda_{2}}{\mu_{2}-\mu_{1}+\lambda_{1}}\ll 1.$$

We remark that as a special case, if $x\equiv \frac{\lambda_{2}}{\mu_{2}-\mu_{1}}\ll 1$, then the above condition will hold (since $\lambda_{1}\geq 0$), and active protocells will dominate. However, the inequality (A9) gives a more general condition for active protocell domination.
