# Supplementary material for: Multistable Protocells Can Aid the Evolution of Prebiotic Autocatalytic Sets
Source: Life (Basel). 2023 Dec 12;13(12):2327. doi: 10.3390/life13122327 (PMC10744544; doi:10.3390/life13122327)
Supplement: Supplementary file 1 [file life-13-02327-s001.zip › life-2635562-supplementary.pdf]

Supplementary Material

Title of paper: Multistable protocells can aid the evolution of prebiotic autocatalytic sets
Authors: Angad Yuvraj Singh and Sanjay Jain

Contents

1 Robustness of the results to changes in the model structure **2**

2 Robustness of model behaviour at other values of catalytic efficiency (κ) **7**

2.1 $\kappa = 2000$ 7

2.2 $\kappa = 3400$ 8

3 Comparison of f from mean field model with f obtained by simulations **9**

4 Data structure and cleaning methodology **11**

4.1 Stochastic single cell growth-division 11

4.2 Population of protocells 12

1 Robustness of the results to changes in the model structure

In this section we present results for a protocell model with five chemical species that relaxes certain constraints and assumptions of the model presented in the main paper in order to show the robustness of the results of the main paper. The five species include two monomers $A(1)$ and $B(1)$, two dimers $A(2)$ and $B(2)$, and one tetramer $A(4)$. Their respective populations in the protocell are denoted X_1, Y_1, X_2, Y_2 and X_4. The main differences are as follows:

- The rate of intake of food molecules is proportional to their difference in concentration between the outside and inside of the protocell.
- There are two types of monomers, $A(1)$ and $B(1)$, both treated as food molecules, instead of just one.
- In the model presented in the main paper, the dimer $A(2)$ was doing double duty as the enclosure forming molecule as well as a reactant to form the catalyst $A(4)$. Here the two roles are performed by different molecules, the enclosure forming molecule being the dimer $B(2)$.
- The definition of the protocell volume excludes the population of the enclosure forming molecule (only includes populations of molecules in the bulk of the protocell), as an example of an alternate linear combination of chemical populations.

While the quantitative outcomes depend upon the details, the qualitative results remain the same. These include the presence of bistability in a robust parameter region, two distinct growth rates for the two attractors, and selection of the state where the ACS is active.

The reaction scheme is as follows:

$$
\begin{aligned}
&\textbf{Transport of } A(1): && A(1)_{ext} \xrightarrow{\alpha Y_2} A(1) \\
&\textbf{Transport of } B(1): && B(1)_{ext} \xrightarrow{\alpha Y_2} B(1) \\
&\textbf{R1 (uncatalyzed)}: && 2A(1) \underset{k_R}{\overset{k_F}{\rightleftharpoons}} A(2) \\
&\textbf{R1 (catalyzed)}: && 2A(1) + A(4) \underset{\kappa k_R}{\overset{\kappa k_F}{\rightleftharpoons}} A(2) + A(4) \\
&\textbf{R2 (uncatalyzed)}: && 2A(2) \underset{k_R}{\overset{k_F}{\rightleftharpoons}} A(4) \\
&\textbf{R2 (catalyzed)}: && 2A(2) + A(4) \underset{\kappa k_R}{\overset{\kappa k_F}{\rightleftharpoons}} A(4) + A(4) \\
&\textbf{R3 (uncatalyzed)}: && 2B(1) \underset{k_R}{\overset{k_F}{\rightleftharpoons}} B(2) \\
&\textbf{R3 (catalyzed)}: && 2B(1) + A(4) \underset{\kappa k_R}{\overset{\kappa k_F}{\rightleftharpoons}} B(2) + A(4) \\
&\textbf{Degradation}: && A(2) \xrightarrow{\phi} \emptyset,\ A(4) \xrightarrow{\phi} \emptyset,\ B(2) \xrightarrow{\phi} \emptyset.
\end{aligned}
$$

In this model, the enclosure is formed by the dimers of the type $B(2)$ and is permeable only to the monomers $A(1)$ and $B(1)$. The rates at which monomers diffuse into the interior of the protocell is taken to be proportional to the number of $B(2)$ and the difference in the monomer concentrations inside and outside, α being the proportionality constant. The three catalyzed reactions **R1, R2, R3**, all catalyzed by $A(4)$, together with the two transport reactions, form an autocatalytic set. The enclosure forming molecule $B(2)$ may be considered effectively a catalyst for the transport reactions. The deterministic system of equations for this model is:

$$
\frac{dX_1}{dt} = \alpha Y_2(x_1^{ext} - \frac{X_1}{V}) - 2(1 + \kappa\frac{X_4}{V})(\frac{k_F X_1^2}{V} - k_R X_2) \tag{1}
$$
$$
\frac{dX_2}{dt} = (1 + \kappa\frac{X_4}{V})(\frac{k_F X_1^2}{V} - k_R X_2) - 2(1 + \kappa_4\frac{X_4}{V})(\frac{k_F X_2^2}{V} - k_R X_4) - \phi X_2 \tag{2}
$$
$$
\frac{dX_4}{dt} = (1 + \kappa\frac{X_4}{V})(\frac{k_F X_2^2}{V} - k_R X_4) - \phi X_4 \tag{3}
$$
$$
\frac{dY_1}{dt} = \alpha Y_2(y_1^{ext} - \frac{Y_1}{V}) - 2(1 + \kappa\frac{X_4}{V})(\frac{k_F Y_1^2}{V} - k_R Y_2) \tag{4}
$$
$$
\frac{dY_2}{dt} = (1 + \kappa\frac{X_4}{V})(\frac{k_F Y_1^2}{V} - k_R Y_2) - \phi Y_2, \tag{5}
$$

where x_1^{ext} and y_1^{ext} are the fixed monomer concentrations outside the protocell. The volume for this model is defined as $V = v(X_1 + Y_1 + 2X_2 + 4X_4)$. This definition of V excludes the

population of $B(2)$. (One might imagine that $B(2)$ is a lipid molecule; once produced inside the protocell it immediately migrates to the boundary and becomes part of the enclosure, and is therefore excluded from the bulk of the protocell.) As in the main paper the protocell is assumed to divide into two equal daughters when its volume reaches the upper limit V_c.

We now present the behaviour of this model along the same lines as the model presented in the main paper, and show that the 5-chemical species model has the same kind of dynamics at both the single protocell and the ecosystem-of-protocells levels as the simpler model presented in the main paper. Here too there is a bistability with the ACS active protocells having a much higher growth rate than the ACS inactive ones; see Fig. 1 in the Supplementary Material (SM). Under stochastic chemical dynamics the ACS can arise by chance in a single protocell that initially has no ACS (see Fig. 2 in SM), and then the ACS active cells can take over and dominate the population of protocells (see Fig. 3 in SM). This shows the robustness of the behaviour exhibited by the model in the main paper.

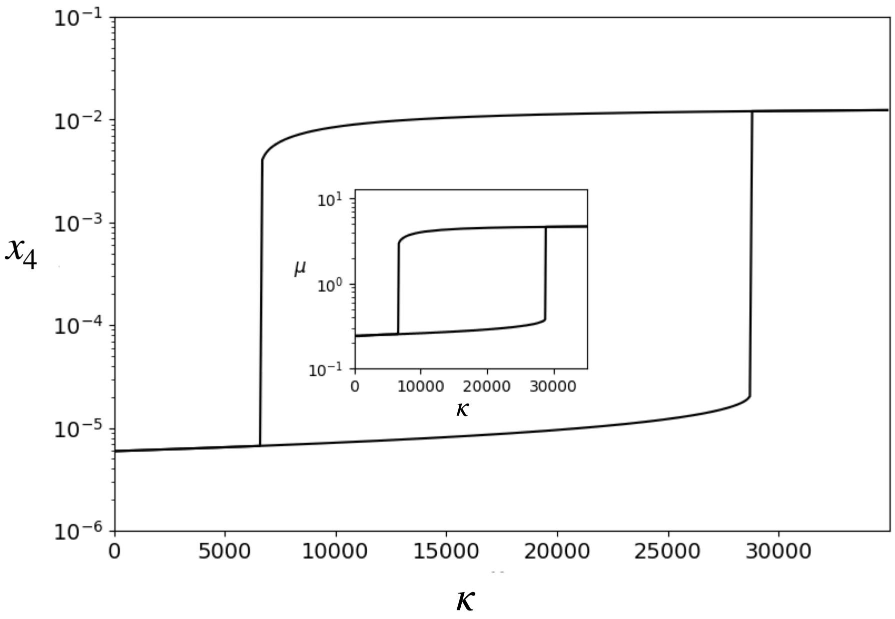

Figure 1: Bifurcation diagram for the 5-chemical-species model. Parameters: $k_F = k_R = v = 1$, $\phi = 20$, $\alpha = 100$. External concentrations: $x_1^{ext} = y_1^{ext} = 1$. In the upper branch (ACS active) the catalyst has a concentration that is about three orders of magnitude higher than the lower branch (ACS inactive). The inset shows that the growth rate of the protocell in the ACS active state is about one order of magnitude higher than in the ACS inactive state.

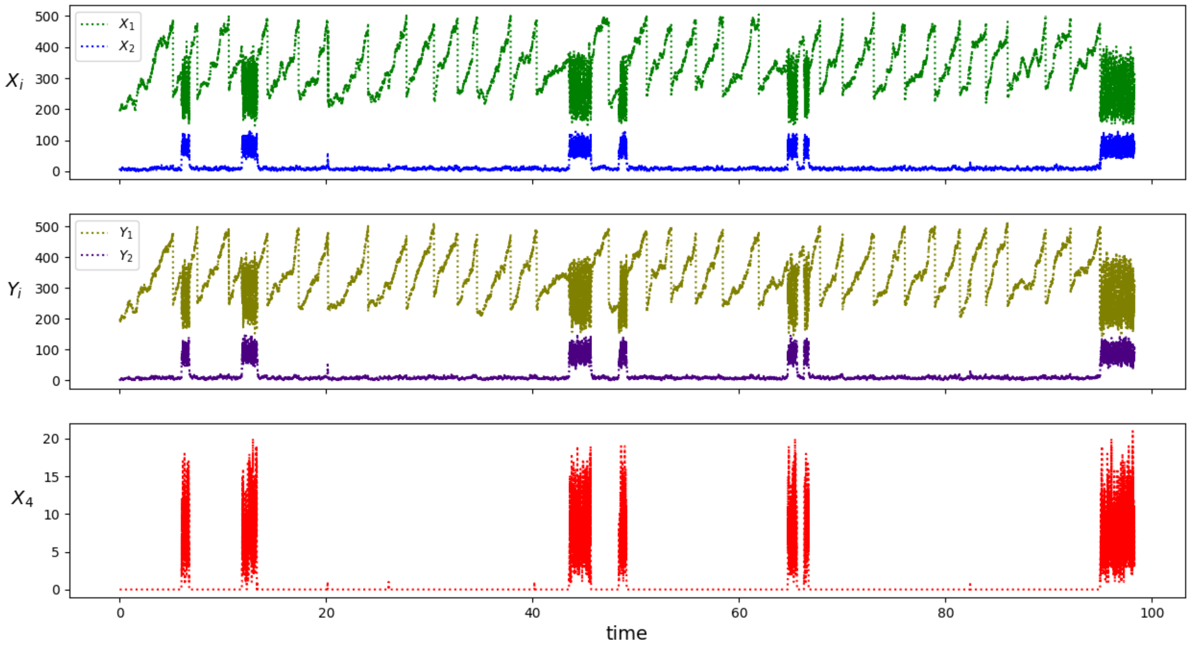

Figure 2: Stochastic simulation of population of species A(1), B(1), A(2), B(2) and A(4) for the model using the Gillespie algorithm. Each reaction has a probability of occurrence per unit time that is related to the deterministic reaction rate along the same lines as given in Table A1 of Appendix A for the model in the main paper. Parameter values: $\kappa = 15000$; rest same as in Fig. 1 of Supplementary Material. Initial condition: $X_1 = Y_1 = 200$, $X_2 = Y_2 = 10$, $X_4 = 0$. From a long such simulation we find that the average interdivision times in the inactive and active states are, respectively, $\langle \tau_1 \rangle = 2.768$, $\langle \tau_2 \rangle = 0.164$, while the average residence times in the two states are $\langle T_1 \rangle = 20.728$, $\langle T_2 \rangle = 3.058$.

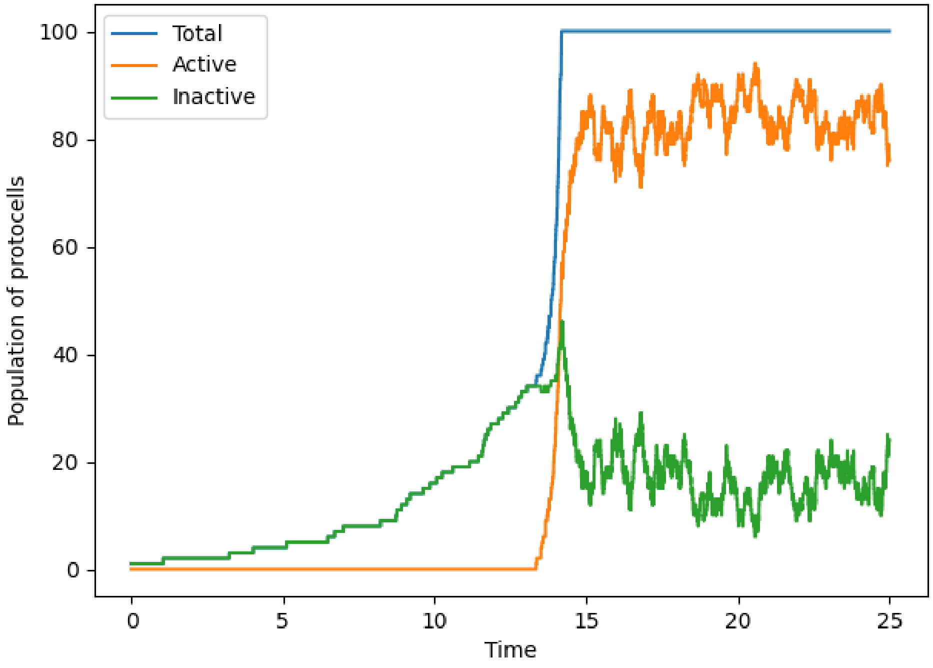

Figure 3: Time evolution of a population of protocells in the 5-chemical-species model starting from a single protocell in the inactive state. Each individual protocell is simulated by the Gillespie algorithm for its internal chemical dynamics. Shown is the number of protocells in the inactive state (green), active state (orange), and their sum (blue). After the total population reaches an externally imposed ceiling (100 in this figure), upon each further cell division a randomly chosen protocell is removed from the population. Parameters: $\kappa = 15000$, $k_F = 1$, $\phi = 20$, $\alpha = 100$, $V_c = 1000$. Note the domination of the active protocell population in the stochastic steady state of the protocell population dynamics, starting from an initial state with only one protocell in the inactive state.

2 Robustness of model behaviour at other values of catalytic efficiency (κ)

In this section we show the model behaviour at two other values of the catalytic efficiency κ closer to the two ends of the bistable region of κ shown in Fig 2 of the main paper, keeping all the other parameters the same as used to generate plots in the main paper, i.e., $k_F = 1.0,\ \phi = 20,\ \alpha = 100,\ V_c = 1000$.

2.1 $\kappa = 2000$

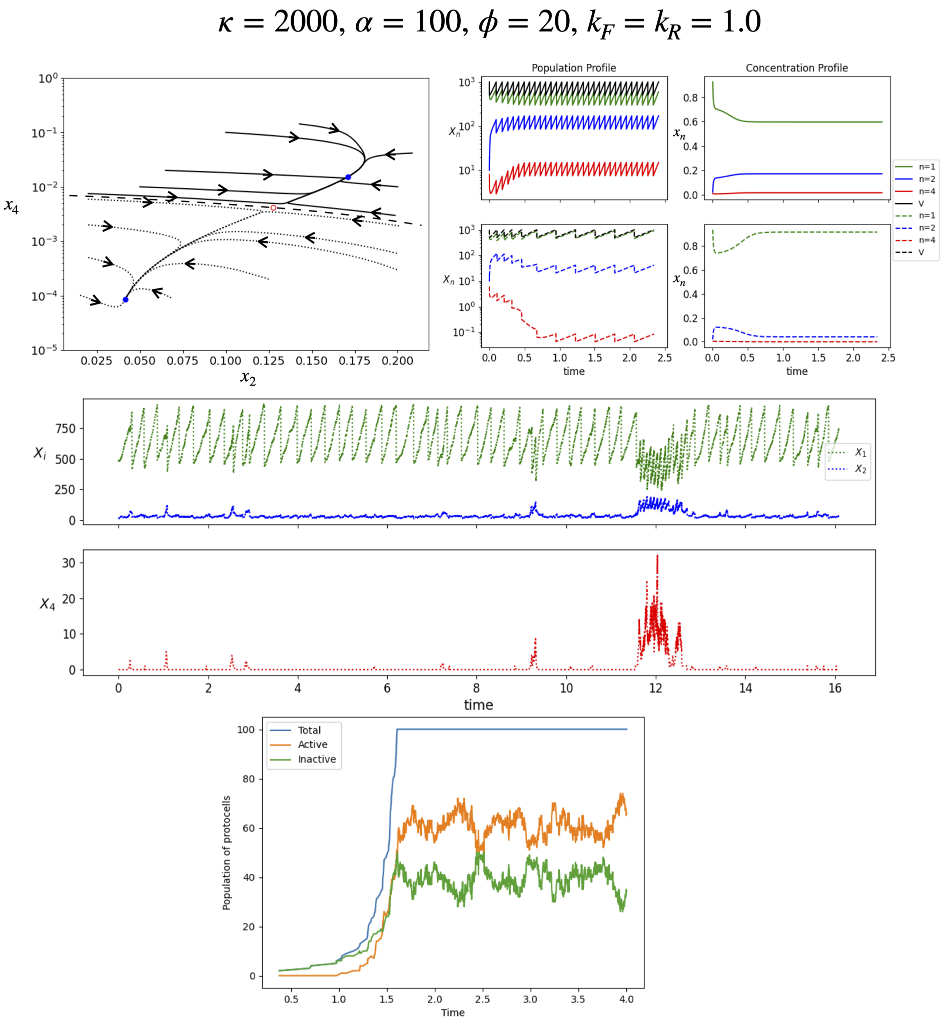

Figure 4: Simulations of the model at $\kappa = 2000$. The same panels as in Figs. 3, 4 and 5 of the main paper are shown, but at $\kappa = 2000$. For the deterministic case, concentrations of the molecules as a function of time are also shown.

2.2 $\kappa = 3400$

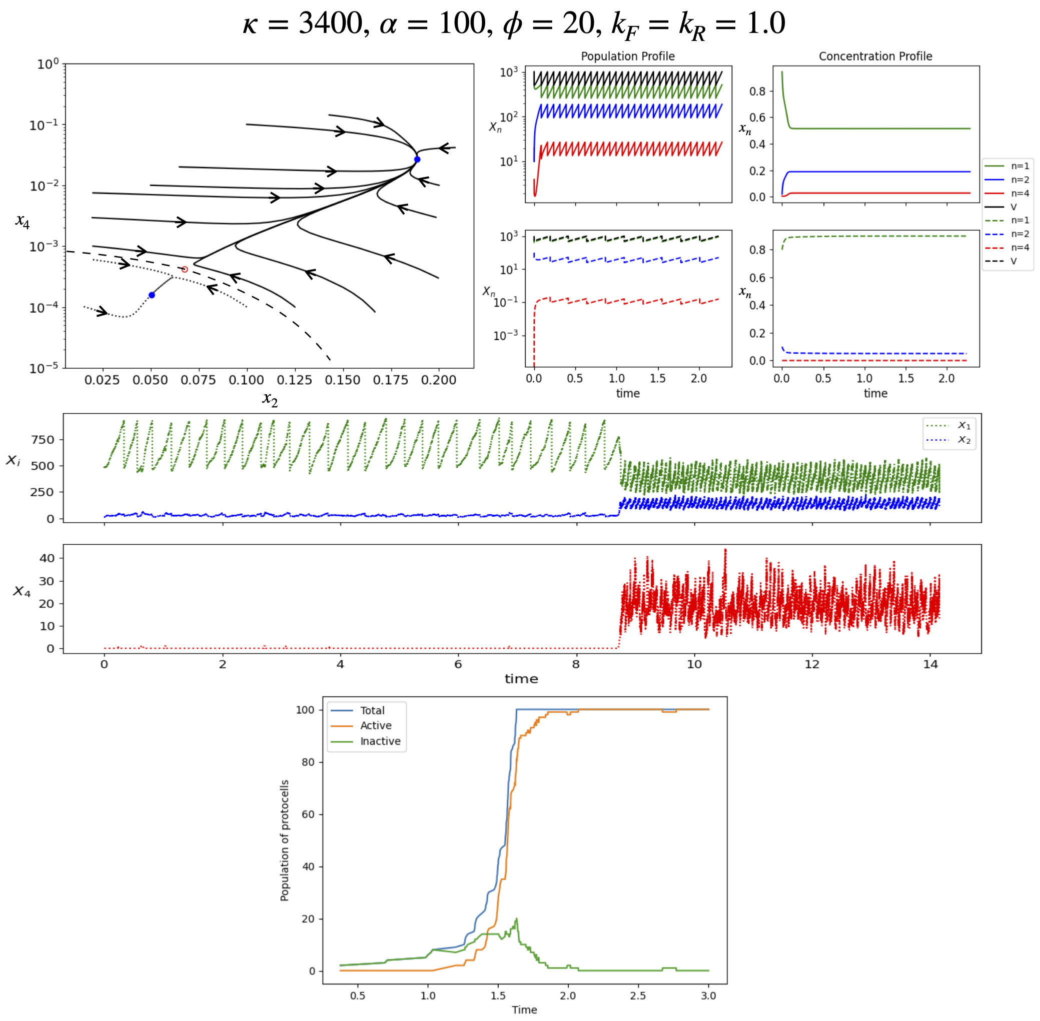

Figure 5: Simulations of the model at $\kappa = 3400$. The same panels as in Figs. 3, 4 and 5 of the main paper are shown, but at $\kappa = 3400$. For the deterministic case, concentrations of the molecules as a function of time are also shown.

As κ increases within the bistable region, the lifetime of the inactive state decreases and that of the active state increases. This is expected since the basin size of the inactive attractor declines and that of the active attractor grows as κ increases from κ^I to κ^{II} (see, e.g., the difference between the unstable branch and the two stable branches in Fig. 2 of the main paper). This increases the steady state fraction of the active protocells in the dynamics of protocell populations. However the qualitative behaviour of the model is unchanged.

κ	$\mu_1 \pm \Delta\mu_1$	$\mu_2 \pm \Delta\mu_2$	$\lambda_1 \pm \Delta\lambda_1$	$\lambda_2 \pm \Delta\lambda_2$	$f \pm \Delta f$	$f_{sim} \pm \Delta f_{sim}$
1900	2.371 ± 0.013	8.508 ± 0.684	0.072 ± 0.011	3.122 ± 0.423	0.503 ±0.122	0.475 ± 0.05
2000	2.372 ± 0.013	8.752 ± 0.075	0.096 ± 0.015	2.205 ± 0.351	0.662 ± 0.059	0.664 ± 0.027
2200	2.324 ±0.013	8.861 ± 0.045	0.179 ± 0.025	1.074 ± 0.152	0.841 ± 0.024	0.854 ± 0.023
2400	2.352 ± 0.017	8.976 ± 0.031	0.293 ± 0.035	0.522 ± 0.092	0.925 ± 0.014	0.937 ± 0.019
2600	2.290 ± 0.020	9.081 ± 0.028	0.300 ± 0.042	0.314 ± 0.086	0.956 ± 0.013	0.974 ± 0.012
2800	2.277 ± 0.034	9.112 ± 0.027	0.359 ± 0.090	0.144 ± 0.030	0.980 ± 0.005	0.992 ± 0.006

Table 1: Comparison of f from mean field model and stochastic simulations of protocell population dynamics (f_{sim}) for different values of κ. Parameters: $v = k_R = 1$, $k_F = 1$, $\phi = 20$, $\alpha = 100$, $V_c = 1000$. For calculating f from the mean field model, the parameters μ_1, μ_2, λ_1, and λ_2 are estimated as discussed in the main paper as well as in Appendix B of the main paper. f_{sim} data was generated from stochastic simulations of protocell dynamics in which the ceiling of the total number of protocells was taken to be $K = 250$.

3 Comparison of f from mean field model with f obtained by simulations

Table 1 and Fig. 6 of the supplementary material compare the value of f obtained from the analytic expression given in Eq. (13) of main paper and in Appendix C with the value in stochastic simulations of the protocell dynamics discussed in the main paper (denoted f_{sim}), at different values of the catalytic efficiency κ.

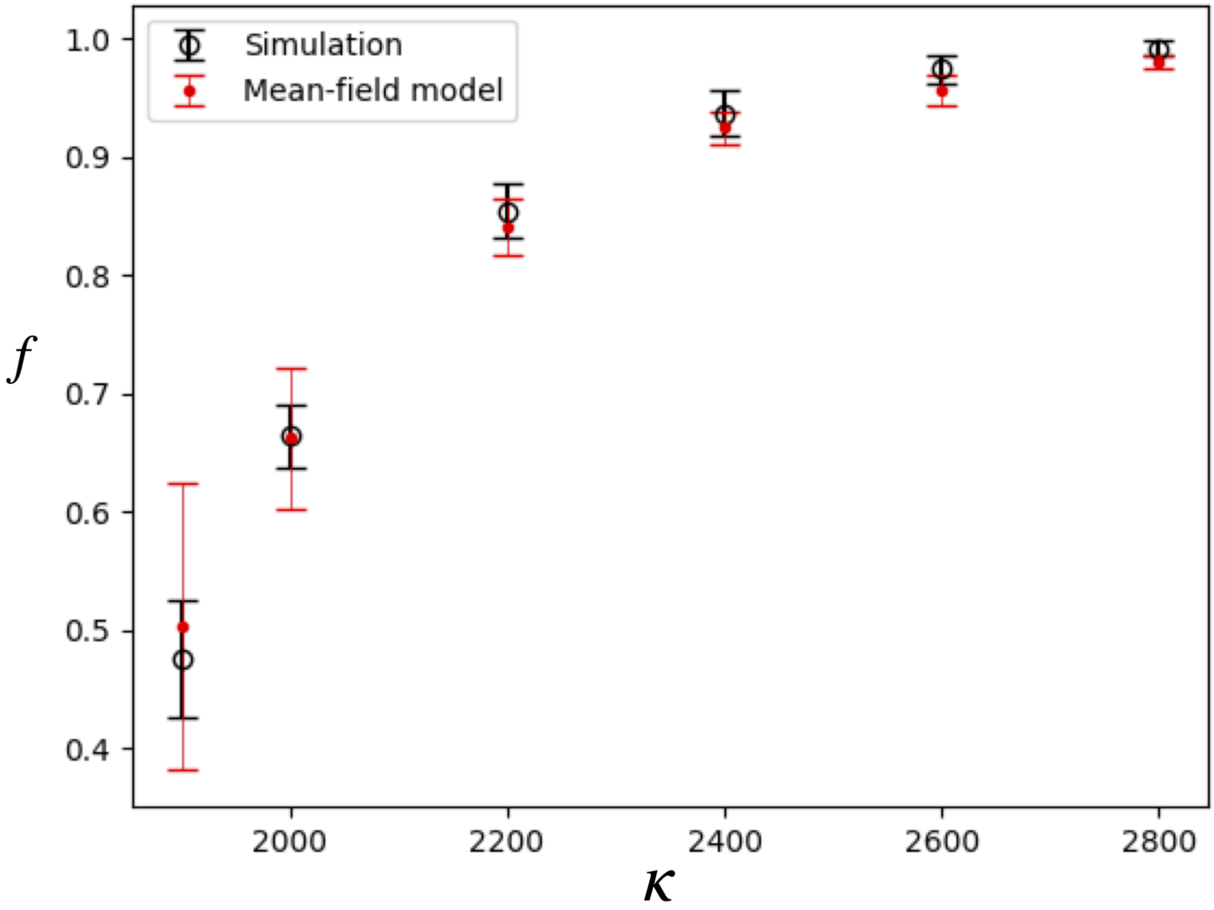

Figure 6: Fraction f of 'ACS active' protocells in the stochastic steady state of the protocell population dynamics versus κ, obtained from simulation (black hollow circles) and the mean field model (red solid dots). The error bars in f and f_{sim} are $\pm\Delta f$ and $\pm\Delta f_{sim}$ respectively, whose calculation is discussed in Section 3 of the Supplementary Material. Data taken from Table 1 of Supplementary Material. $k_F = 1,\ \phi = 20,\ \alpha = 100,\ V_c = 1000$.

The analytic value of f obtained from the mean field model agrees with f_{sim} within error bars. Note that the individual parameters μ_1, μ_2, λ_1, and λ_2 in the analytic expression for f are obtained from average values of τ_1, τ_2, T_1 and T_2 calculated from the respective histograms (such as those displayed in Fig. A1 of Appendix B in the main paper) generated from the stochastic simulation of a single growing and dividing protocell. Therefore, all of the parameters have errors (given in Table 1) arising from the standard errors of the means. The error Δf in f is computed from the analytical expression of f using the above mentioned standard errors in each of the four quantities. The error Δf_{sim} in f_{sim} is just the standard deviation of f_{sim} in the stochastic steady state.

4 Data structure and cleaning methodology

Data analysis was primarily performed using data generated from two stochastic simulations: **Stochastic single cell growth-division** and **Population of protocells**.

4.1 Stochastic single cell growth-division

Following is the format of data prepared for analysing various aspects of the model:

1. **Raw Data Level 0:** Raw data is first stored in the structure given in Table 2. The raw data consists of the copy number of species (X_i), time at which the reaction occurred, volume of the protocell, and the generation at which the cell is when the internal reactions are happening. The generation count[1] is set to 0 at the start of the simulation.

Generation count	Time of reaction	V	X_1	X_2	X_4

Table 2: Representation of data generated for Raw Data Level 0.

2. **Raw Data Level 1:** From the Raw Data Level 0, data **ONLY** at the time of division is extracted and stored separately in the format given in Table 3. This data has the values of species copy number and the state (in terms of binary string '0' for inactive or '1' for active) of the mother protocell at the time of division.

Generation count	State (0/1)	Time at division	V	X_1	X_2	X_4

Table 3: Representation of data generated for Raw Data Level 1.

Note that the time is recorded only at the point when the cell divides. This time marks the end of previous generation or start of the next generation. A transition can occur at any point within a single cell cycle. However, the state of the cell (0 or 1) is noted only at the time of division in the data above. The state of the cell is decided as per the criteria defined in Appendix B in the paper. Transient from this data is removed as per the guidlines given next.

[1]Generation count is defined as the number of divisions the cell has undergone during the course of the simulation.

3. **Removing the transient**: The transient trajectory of the cell is defined as the initial phase where the concentrations of the species inside the cell have not reached a stochastic steady state. To generate the data set to extract the parameters of the mean field model (residence times and interdivision times) for the two states from a run, the transient in the beginning of the run needs to be removed from data stored in Raw Data Level 1. It is typically observed that the stochastic steady state is reached within the first few division cycles. After 15 division cycles we ask: Has a transition occurred yet? There could be two possibilities.

   (a) **No transition has taken place in the first 15 division cycles:** Then the point where the first transition occurs is the start of data recording. This point is the first instance where the cell has changed its state (from 0 to 1 or 1 to 0).

   (b) **A transition has taken place in the first 15 division cycles:** In this case, the first transition is ignored. Data collection starts from the second transition point irrespective of whether it is within the first 15 division cycles or not.

4. **Truncating data collection**: Data collection stops at the last transition point in the run. This point is the last instance where the cell changes its state (from 0 to 1 or 1 to 0).

The Level 1 data modified by the removal of the initial transient and truncation of the end of the run is stored separately in same format as given in Table 3, and used to construct the histograms of single protocell parameters τ_1, τ_2, T_1, T_2.

4.2 Population of protocells

In this simulation, data is generated in two formats:

1. **Data of individual protocell:** Each time a protocell divides, a new file is generated storing the data of one of the daughter protocells starting with the population of chemical species at birth in the format shown in Table 4 while the data of the other daughter cell is appended to the mother cell file. Each time a reaction occurs in any cell, the revised species count (X_i) is appended to the file corresponding to that cell. The total number of files is equal to the number of division cycles plus number of cells the simulation began with [2].

[2]For results shown in the paper, the simulation started with a single cell but one can also run the simulation starting with n cells

State (0/1)	Time of reaction	V	X_1	X_2	X_4

Table 4: Representation of data generated for a particular cell in the protocell population simulation.

2. **Summary Data**: A summary file is created that stores the number of active/inactive protocells at every division by extracting data of all the existing protocells at the division time points. This information is stored in the format given in Table 5. This data is used to generate Fig. 5 of the main paper.

Total no. of cells	Division Time	No. of Active cells	No. of Inactive Cells

Table 5: Format of the data stored in file containing the number of active/inactive protocells in the population.
